# Effects of Variation in *Tamarix chinensis* Plantations on Soil Microbial Community Composition in the Middle Yellow River Floodplain

**DOI:** 10.3390/ijerph20065015

**Published:** 2023-03-12

**Authors:** Xinyu Yan, Lanlan Zhang, Qi Xu, Linyu Qi, Jingyuan Yang, Xiongde Dong, Meiguang Jiang, Mengjun Hu, Junqiang Zheng, Yanyan Yu, Yuan Miao, Shijie Han, Dong Wang

**Affiliations:** International Joint Research Laboratory for Global Change Ecology, School of Life Sciences, Henan University, Kaifeng 475004, China

**Keywords:** microbial community composition, resource islands, soil microbial biomass, *Tamarix chinensis* Lour., the middle Yellow River floodplain, vegetation pattern

## Abstract

Floodplains have important ecological and hydrological functions in terrestrial ecosystems, experience severe soil erosion, and are vulnerable to losing soil fertility. *Tamarix chinensis* Lour. plantation is the main vegetation restoration measure for maintaining soil quality in floodplains. Soil microorganisms are essential for driving biogeochemical cycling processes. However, the effects of sampling location and shrub patch size on soil microbial community composition remain unclear. In this study, we characterized changes in microbial structure, as well as the factors driving them, in inside- and outside-canopy soils of three patch sizes (small, medium, large) of *T. chinensis* plants in the middle Yellow River floodplain. Compared with the outside-canopy soils, inside-canopy had higher microbial phospholipid fatty acids (PLFAs), including fungi, bacteria, Gram-positive bacteria (GP), Gram-negative bacteria (GN), and arbuscular mycorrhizal fungi. The ratio of fungi to bacteria and GP to GN gradually decreased as shrub patch size increased. Differences between inside-canopy and outside-canopy soils in soil nutrients (organic matter, total nitrogen, and available phosphorus) and soil salt content increased by 59.73%, 40.75%, 34.41%, and 110.08% from small to large shrub patch size. Changes in microbial community composition were mainly driven by variation in soil organic matter, which accounted for 61.90% of the variation in inside-canopy soils. Resource islands could alter microbial community structure, and this effect was stronger when shrub patch size was large. The results indicated that *T. chinensis* plantations enhanced the soil nutrient contents (organic matter, total nitrogen, and available phosphorus) and elevated soil microbial biomass and changed microbial community composition; *T. chinensis* plantations might thus provide a suitable approach for restoring degraded floodplain ecosystems.

## 1. Introduction

Floodplains, located in the transition zone between aquatic and terrestrial ecosystems, are characterized by high primary productivity and biological diversity [1,2]. Due to the interference of climate change and human activities, floodplain ecosystem stability has been seriously disturbed, and this has resulted in soil erosion and land degradation [3,4]. Aiming at preventing soil erosion, shrub vegetation restoration projects are more extensive and are an effective method to combat land degradation than herb and tree in floodplain ecosystems [5]. Shrub plantation drives changes in soil physiochemical properties, soil organic matter (SOM) formation, and pyrolysis as well as vegetation structure composition and plant biomass allocation pattern [4,6,7]. All the changes have profound impacts on soil microbial community composition and activity [7,8].

Soil microorganisms play critical roles in nutrient cycling, carbon sequestration, and ecosystem stability in terrestrial ecosystems [8,9]. Soil microbial biomass, community composition, and physiological activities are sensitive to aboveground vegetation and belowground conditions [9]. Root-associated arbuscular mycorrhizal fungi acquired substantial amounts of nitrogen and phosphorous for plant uptake from decomposed organic matter [10,11]. Bacteria are essential for affecting nutrient availability and transforming plant-derived and microbial-derived biomass into soil organic matter [12]. Shrubs can maintain ecosystem functions by forming fertile islands beneath their canopies [7]. Indeed, *T. chinensis* (*Tamarix chinensis* Lour.) can grow in various shrub patch sizes, and soil properties and soil microbial community structure vary with shrub patch size [13].

Variation in soil microbial structure is controlled by various biotic and abiotic factors [9,14]. Shifts in the quantity and quality of carbon due to variations in plant communities affect the composition and function of microbial communities [15]. Resource availability (e.g., water, soil organic carbon, and phosphorus) has a strong effect on the soil microbial community composition [16,17]. In the nutrient fertilization experiment, spatial heterogeneity in the distribution of soil organic matter alleviates microbial carbon limitation [18]. Moreover, shrub patch size has a significant effect on soil organic carbon, total nitrogen, and soil microbial biomass [15,19]. The fungal abundance and biomass of *Artemisia gmelinii* are higher in inside-canopy soils than in outside-canopy soils due to the increased SOC input in semiarid land [20]. The ability of shrubs to alter the accumulation of nutrients has been demonstrated in desert-wash shrub communities, shrub-dominated semi-desert ecosystems, semi-arid savanna, and alpine ecosystems [21,22,23,24]. However, the effects of patch size and sampling location around individual shrub plants on the relationship between soil microbial community structure and soil physicochemical properties have not been thoroughly examined.

*Tamarix chinensis* Lour., northern Tamarisk, a shrub species with high coverage and high tolerance to soil erosion and land degradation, is a vital part of the ecological project of “Southern Mangrove and Northern Tamarisk” [25,26]. This species is widely distributed in the middle Yellow River floodplain [27]. The middle Yellow River floodplain is derived from the Loess Plateau and experiences severe soil erosion; nutrients in this region are thus being depleted from its fine-grained soils [28,29]. Shrub patch size can affect the dynamics of soil physiochemical properties and alter soil microbial community composition [15,18,19]. In turn, soil microorganisms such as bacteria and fungi can affect soil nutrient cycling processes by catalyzing decomposition of litter and SOM [10,11]. Significant research has mainly focused on the soil properties under different shrub plantations [21,22,23,24], but the dynamics of microbial community composition under the *T. chinensis* plantations remain unclear. To a certain extent, the data deficiency has hindered the construction of *T. chinensis* shelter forest in the floodplain. In this paper, we investigated the effects of *T. chinensis* plantation of different shrub patch sizes and sampling locations on soil physicochemical properties and soil microbial community composition in the middle Yellow River floodplain. This study was motivated by two questions: (1) How do the variations of microbial community composition change through shrub patch size and sampling location following the *T. chinensis* plantation? (2) What were the underlying mechanisms influencing the responses of microbial community activities to *T. chinensis* plantation in the middle Yellow River floodplain?

## 2. Materials and Methods

### 2.1. Study Region Description

**Figure 1:**
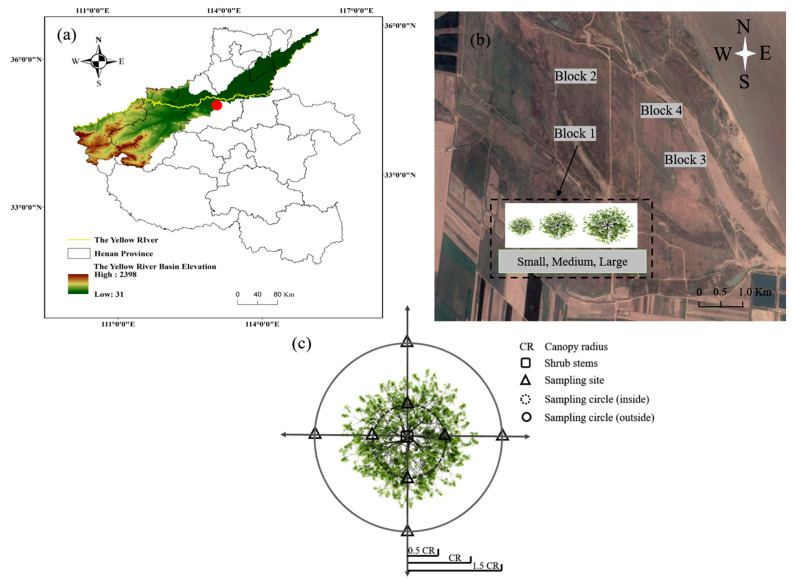
Maps and schematic diagram of the sampling location site in the Yellow River floodplain of Henan Province, China. (**a**) Color covering area is the Yellow River basin; yellow line is the main channel of Yellow River; red dot is the sample area in the Yellow River floodplain. (**b**) The map indicates the sample sites in the Yellow River floodplain. (**c**) The location of the sample site. CR represents the canopy radius. The square is the stem of *T. chinensis*. The triangle represents the sampling site inside and outside of the canopy. The circle represents the sampling range inside and outside of the canopy.

This study was conducted at the Yellow River Floodplain Ecosystems Research Station (34°59′65″ N, 113°25′05″ E, 100 m a.s.l.), Xingyang County, Henan Province, China (Figure 1a). The region experiences a monsoon climate. The mean annual average temperature was 14.3 °C; the highest monthly mean temperature was 42.5 °C (in July), and the lowest monthly mean temperature was −9.6 °C (in January). The mean annual precipitation was 645.5 mm, and 65% of the precipitation fell between July and September. The soil at the study site was sandy, and the pH was approximately 8.10 [30]. The vegetation is dominated by *T. chinensis*, *Phragmites australis* (Cav.) Trin., *Cynodon dactylon* (L.) Pers., *Calamagrostis epigeios* (L.) Roth., and *Aster subulatus* (Michx.) Nesom. [27].

### 2.2. Experimental Design and Sampling

*T. chinensis* is an important component of the vegetation, and it grows in individual clumps of plants up to 300 cm across the canopy in the Middle Yellow River floodplain according to our survey data. This experiment used a randomized block design with four sites (20 m × 20 m) selected, which included the three sizes (small: <100 cm; medium: 100–200 cm, and large: >200 cm) in each site according to the canopy diameter, height, and number of stems of plants in late May 2021 (Figure 1b; Table 1). We selected 12 *T. chinensis* plants for the three size categories, with four repeats for each size. Soil samples were collected along horizontal transects on two different radii surrounding the base of each *T. chinensis* stem (Figure 1c). The soil sampling locations were: (1) at the edge of middle radius of the canopy (inside canopy) and (2) at the edge of one and a half radius of the canopy (outside canopy). The mid-canopy samples were the most appropriate to represent soil physicochemical properties under canopies [31]. The outside samples were at half a canopy radius beyond the drip line to avoid influences from neighboring shrubs [32]. At a 0–15 cm soil depth, four soil samples in each radius were collected by a 4 cm inner diameter soil auger in East, South, West, and North, respectively, and then composited into one sample (Figure 1) [7,33]. There were, in total, 24 mixed soil samples which were passed through a 2 mm sieve and placed in sterile plastic pouches. Each sample was divided into two parts: one part of the sample was air-dried, ground, and used for the determination of soil physical and chemical properties, and the other part was stored in iceboxes for microbial analyses.

### 2.3. Analysis of Soil Properties

Soil particle size analyses were conducted using the laser diffraction technique with a Longbench MasterSizer 2000 (Malvern Instruments, Malvern, England, UK) to calculate the percentage of clay, silt, fine sand, and coarse sand. The soil water content (SWC, g of water per 100 g of dry soil) was determined by oven drying (ZXRD_A7230, Zhicheng, Shanghai, China) samples at 105 °C to a constant weight and then taking weight measurements. Soil pH was measured using a pH meter (Sartorius PB-10, Göttingen, Germany) with a 2.5:1 ratio of deionized water/air-dried soil. The soil salt content (SSC) was determined using the gravimetric method [34]. Soil organic carbon (SOC) was analyzed using dichromate-sulfuric acid oxidation with heating. SOC was converted to soil organic matter (SOM) by multiplying with the constant of 1.724 [35]. Soil total nitrogen (TN) was measured using a Vario Max CNS elemental analyzer (Elementar Analysensysteme GmbH, Hanau, Germany). Available phosphorus (AP) was extracted with 0.5 M NaHCO3; measurements were then taken using the UV Spectrophotometer (Daojin UV-1900, Kyoto, Japan).

### 2.4. Phospholipid Fatty Acid Analysis

Soil microbial community composition was characterized by analyzing PLFAs [36]. Briefly, lipids were extracted from soil with a chloroform: methanol: 0.05 M sodium phosphate buffer mixture (1:2:0.8 (*v*/*v*/*v*)); they were then separated into neutral lipids, glycolipids, and phospholipids using a pre-packed silica column. Phospholipids subjected to mild alkaline methanolysis and fatty acid methyl esters were identified using a gas chromatograph with a flame ionization detector (FID) (GC6890, Agilent Technologies, Bracknell, UK) with methyl nonadecanoate (19:0) as the internal standard. The abundance of individual fatty acid methylesters was expressed as nmol/g dry soil. The total microbial biomass was estimated as the sum of all the extracted PLFAs.

Lipid markers associated with microbial functional groups were analyzed by summing their concentrations. The groups detected included bacteria (15:0, i15:0, i16:0, a16:0, 16:1 *w*6c, a17:0, c17:0, i17:0, 17:1 *w*8c, i17:1 *w*9c, 18:1 *w*5c, 18:1 *w*7c, c19:0 *w*8c, 20:1 *w*9c) [37,38], fungi (16:1 *w*5c, 18:1 *w*9c, 18:2 *w*6,9c) [37,39], Gram-positive bacteria (GP) (a15:0, i15:0, i16:0, a16:0, a17:0, i17:0, i17:1 *w*9c), Gram-negative bacteria (GN) (16:1 *w*6c, c17:0, 17:1 *w*8c, 18:1 *w*5c, 18:1 *w*7c, c19:0 *w*8c, 20:1 *w*9c) [40,41], and arbuscular mycorrhizal fungi (16:1 *w*5c) [37].

### 2.5. Statistical Analysis

All data were expressed as mean ± standard error (SE) of the mean. All variables were transformed to the meet criteria for normality and homoscedasticity, but the results and figures are presented with untransformed values. Two-way analysis of variance was used to assess the effects of shrub patch size, sampling location, and their interaction on soil physicochemical properties and microbial communities. Changes in soil physicochemical properties under the three shrub patch sizes were evaluated using one-way ANOVA, and the differences among sampling locations were evaluated using paired *t*-tests. The least significant difference (LSD) test (*p* < 0.05) was used to identify significant effects. The relationships between microbial communities and soil physicochemical properties were examined using Pearson correlation coefficients. The above statistical analyses were conducted in SPSS 20 (SPSS, Inc., Chicago, IL, USA). Redundancy analysis (RDA) in Canoco 5.0 was performed to determine the environmental factors that affected microbial community composition.

## 3. Results

### 3.1. Shrub Growth Characters and Soil Physicochemical Properties

The basal diameter, height, and canopy diameter of T. chinensis significantly increased with shrub patch size (*p* < 0.001; Table 1). The clay content from small to large shrub patch size changed from 5.54% to 7.82% in inside-canopy soils and from 4.79% to 7.36% in outside-canopy soils. The silt content from small to large shrub patch size altered from 63.65% to 83.59% in inside-canopy soils and from 57.18% to 85.68% in outside-canopy soils. The sand content from small to large shrub patch size varied from 8.58% to 31.76% in inside-canopy soils and from 6.96% to 39.10% in outside-canopy soils (Table 1).

The SSC, SOM, TN, and AP content significantly varied with shrub patch size across the two sampling locations (all *p* < 0.05; Table S1). Averaged across the three shrub patch sizes, the SSC, SOM, TN, and AP were larger in inside-canopy soils than in outside-canopy soils. The SSC, SOM, and TN of inside-canopy soils in the large shrub patch size were 155.47%, 80.33%, and 43.17% higher than the SSC, SOM, and TN of outside-canopy soils in the same shrub patch size, respectively, and these differences were significant. Available *p* was 20.23%, 24.50%, and 54.64% higher in the small, medium, and large shrub patch size in inside-canopy soils than in outside-canopy soils, respectively, and these differences were significant (all *p* < 0.05; Table 2). The SSC, SOM, TN, and AP in inside-canopy soils increased by 86.51%, 75.57%, 38.12%, and 39.03% from the small to large shrub patch size, respectively (all *p* < 0.05; Table 2). The SSC, SOM, and AP in outside-canopy soils increased by 6.15%, 17.42%, and 8.09% from the small to large shrub patch size, respectively, but the TN decreased by 1.19%. There was a strong interaction effect between shrub patch size and sampling location on SSC, SOM, TN, and AP (Table S1). The increases in SSC, SOM, TN, and AP from small to large shrub patch size across both sampling locations were 1.07 g·kg^−1^, 2.62 g·kg^−1^, 0.29 g·kg^−1^, and 14.33 mg·kg^−1^. The value of soil pH decreased with shrub patch size and was lowest under a large patch size in both sampling locations. Neither shrub patch size nor sampling location affected the soil water content.

### 3.2. Effects of Shrub Patch Size and Sampling Location on PLFAs

**Figure 2:**
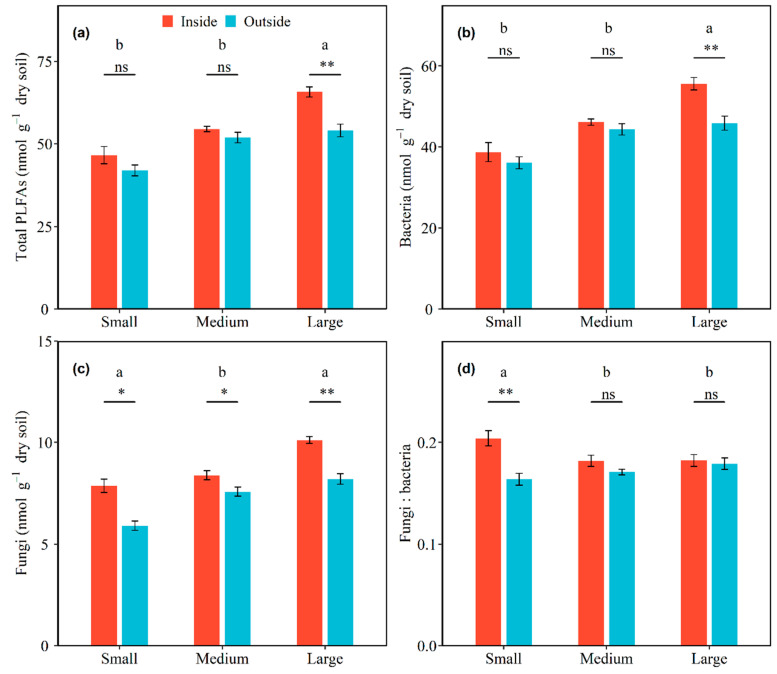
Effects of shrub patch size and sampling location on soil total PLFAs (**a**), bacterial PLFAs (**b**), fungal PLFAs (**c**), and the ratio of fungal to bacterial PLFAs (fungi: bacteria) (**d**). Shrub patches contain small, medium, and large patches, and sampling locations contain inside-canopy and outside-canopy soil. Note: Different letters above the bars indicate significant differences among the different patch sizes of the microbial community changings between inside and outside of plant canopies at the 0.05 level. Symbols above the bars indicate significant differences of each patch size between the inside and outside of plant canopies (** *p* < 0.01, * *p* < 0.05, and ns *p* > 0.05).

**Figure 3:**
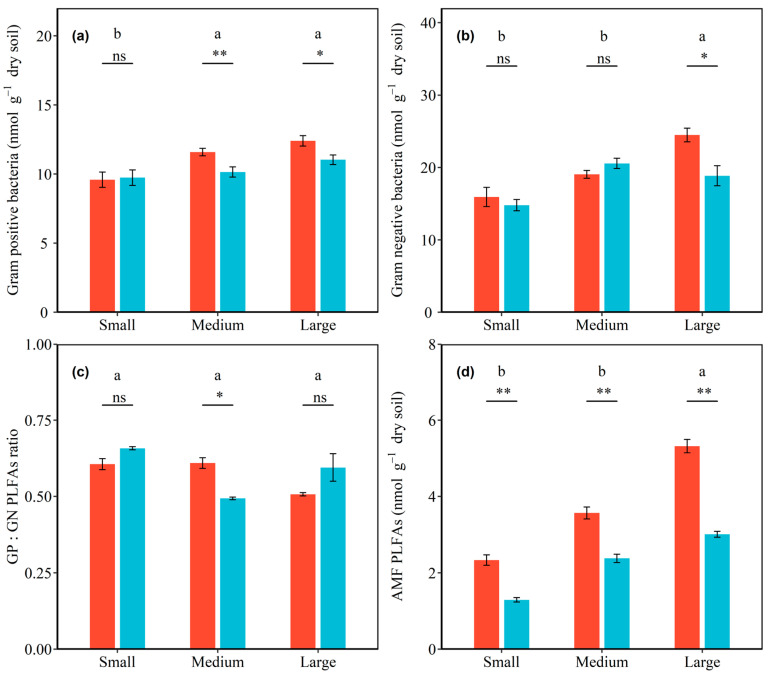
Effects of shrub patch size and sampling location on Gram-positive bacterial PLFAs (**a**), Gram-negative bacterial PLFAs (**b**), the ratio of Gram-positive bacterial PLFAs to Gram-negative bacterial PLFAs (**c**), and arbuscular mycorrhizal fungi (AMF) PLFAs (**d**). Shrub patches contain small, medium, and large patches, and sampling locations contain inside-canopy and outside-canopy soil. Note: The red and blue bars indicate the microbial PLFAs in inside-canopy and outside-canopy soil under three patch sizes. Different letters indicate significant differences among the different patch sizes of the microbial community changings at the 0.05 level. Symbols above the bars indicate significant differences between the inside and outside of plant canopies of each patch size (** *p* < 0.01, * *p* < 0.05, and ns *p* > 0.05).

Microbial community composition varied with shrub patch size and sampling location. Significant interactive effects of total PLFAs, as well as bacterial, fungal, GN bacterial, and AMF PLFAs with shrub patch size and sampling location were detected (all *p* < 0.05; Table S1). In inside-canopy soils, the total PLFAs, as well as bacterial, GP bacterial, GN bacterial, fungal, and AMF PLFAs, were increased by 41.07%, 43.61%, 29.33%, 53.72%, 28.58%, and 127.66% from the small to large shrub patch size, respectively, and these increases were significant. In outside-canopy soils, the total PLFAs, as well as bacterial, GP bacterial, GN bacterial, fungal, and AMF PLFAs, were increased by 28.77%, 27.09%, 13.32%, 27.46%, 39.04%, and 132.43% from the small to large shrub patch size, respectively, and these increases were significant (all *p* < 0.05; Figure 2 and Figure 3; Table 3). The total PLFAs, as well as bacterial, GP bacterial, and GN bacterial PLFAs, were significantly higher in inside-canopy soils than outside-canopy soils in the large shrub patch size (*p* < 0.05; Figure 2 and Figure 3; Table 3). The fungal and AMF PLFAs significantly differed between inside-canopy and outside-canopy soils in all three shrub patch sizes. The fungal PLFAs of inside-canopy soils were 33.42%, 10.69%, and 23.38% higher in the small, medium, and large shrub patch sizes than in outside-canopy soils, respectively. The AMF PLFAs of inside-canopy soils were 80.66%, 49.71%, and 76.94% higher in the small, medium, and large shrub patch sizes than in outside-canopy soils, respectively (*p* < 0.05; Figure 3; Table 3).

There were significant interaction effects between shrub patch size and sampling location on the ratio of fungi to bacteria and GP to GN bacteria (*p* < 0.05; Table S1). The ratio of fungi to bacteria in the inside-canopy soil decreased with shrub patch size, but the ratio of fungi to bacteria in the outside-canopy soils increased with shrub patch size (*p* < 0.05; Figure 2d; Table 3). The ratio of GP to GN bacteria was highest in the small shrub patch size and lowest in the medium shrub patch size in outside-canopy soils (*p* < 0.05; Figure 3c; Table 3).

### 3.3. Correlations between Soil Microbial Community Composition and Physicochemical Properties

**Figure 4:**
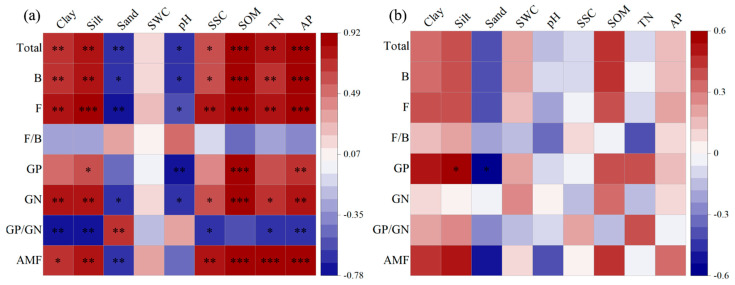
Pearson correlation coefficients between microbial PLFAs and environmental variables inside (**a**) and outside (**b**) of shrub canopies under three patch sizes (SWC: soil water content; SSC: soil salt content; SOM: soil organic matter; TN: soil total nitrogen; AP: available phosphorus; Total: total PLFAs; B: bacterial PLFAs; F: fungal PLFAs; F/B: the ratio of fungal PLFAs to bacterial PLFAs; GP: Gram-positive bacterial PLFAs; GN: Gram-negative bacterial PLFAs; GP/GN: the ratio of Gram- positive bacterial PLFAs to Gram-negative bacterial PLFAs; AMF: arbuscular mycorrhizal fungal PLFAs. Red and blue colors indicate positive and negative correlations, respectively; *** *p* < 0.001, ** *p* < 0.01, * *p* < 0.05).

Pearson correlation analysis showed that total PLFAs, bacterial, fungal, GP bacterial, GN bacterial, and AMF PLFAs were positively correlated with clay, silt, SSC, SOM, TN, and AP but negatively correlated with soil sand and pH (*p* < 0.05). The ratio of GP to GN bacteria was positively related to soil sand (*p* < 0.01), but negatively related to clay, silt, SSC, TN, and AP (*p* < 0.05) in inside-canopy soils under all shrub patch sizes (Figure 4a). In outside-canopy soils, GP bacteria was positively associated with soil silt but negatively associated with soil sand (*p* < 0.05; Figure 4b).

**Figure 5:**
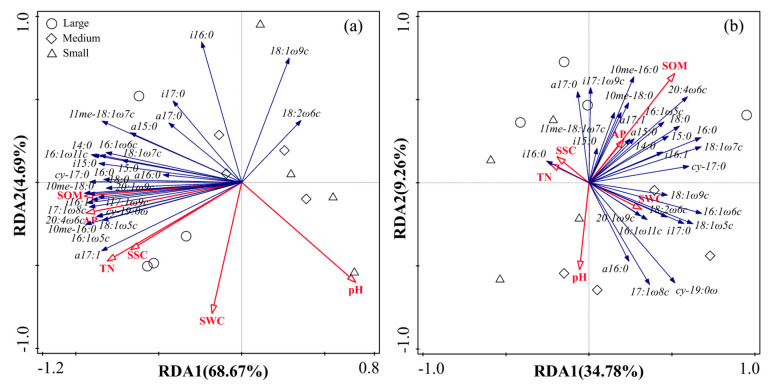
Redundancy analysis (RDA) of soil microbial PLFAs constrained by environmental variables in inside-canopy soil (**a**) and outside-canopy soil (**b**) collected from a depth of 0–15 cm under different shrub patch sizes (large, medium, and small indicate large shrub patch size, medium shrub patch size, and small shrub patch size, respectively).

RDA showed that variation among microbial functional groups was associated with specific soil physicochemical properties (Figure 5). All the physicochemical properties explained 76.10% (axis 1: 68.67%; axis 2: 4.69%) and 48.50% (axis 1: 34.78%; axis 2: 9.26%) of the variance in inside-canopy and outside-canopy soils, respectively. In the inside-canopy soils, the SOM was the most significant variable selected by the forward selection, and it explained 61.90% of the variation in the PLFA data, followed by the AP. The SOM was mainly related to the Gram-positive bacteria PLFA marker (i17:1 *w*9c) and Gram-negative bacteria PLFA marker (17:1 *w*8c, 10me–16:0), and AP was correlated with the AMF PLFA marker (16:1 *w*9c) (*p* < 0.05).

## 4. Discussion

### 4.1. Effect of T. chinensis on Soil Properties

Previous studies demonstrated that the fertile island of shrub enriched soil available nutrients under their canopy, such as SOM, N, and P, supplying more substrates for soil microbes [7,20,42,43]. Here, our results found shrub patch size had a positive effect on soil nutrient status. The SOM, TN, and AP were greater in the inside-canopy soils than in outside-canopy soils under all three shrub patch sizes. The increase in soil nutrients under a canopy may be induced by the microbial decomposition of fallen leaves and root turnover [44]. Aboveground plant litter might be the sources of soil nitrogen, especially under deciduous shrubs, which may enhance nitrogen nutrition [45]. Furthermore, dust particles rich in nitrogen and other mineral elements that accumulate in the leaves and branches could be transported to the soil through stemflow and throughfall [46].

*T. chinensis* generated a “fertile island effect” that could promote the accumulation of soil nutrients, and it tended to increase in strength with shrub patch size [20]. The soil organic matter was 20.61%, 56.43%, and 80.33% higher in the small, medium, and large patch size, respectively, in inside-canopy soils than in outside-canopy soils. The significant difference in soil nutrients between inside-canopy and outside-canopy soils in the three patch sizes indicates that nutrient accumulation occurs over long periods rather than short periods. Increases in soil nutrient conditions might stem from the litter enrichment of shrubs with a larger canopy, which is consistent with previous observations in an arid desert in northwest China [47]. As shrub patch size increased, increases in nutrient availability, especially SOM, under large shrub patch sizes, were mainly attributed to gradual changes in biogeochemical cycles, such as litter decomposition, rhizosphere secretions, and root turnover [47,48]. Furthermore, shifts in soil physicochemical properties among shrub patch sizes and sampling locations might reflect changes in soil microbial communities.

### 4.2. Effect of T. chinensis on Soil Microbial Communities

Soil microorganisms, which have an intricate relationship with soil physicochemical properties, are sensitive to shrub patch size and sampling location [7,9,20]. Previous studies have shown that the growth of vegetated patches has a neutral [49], promotion [20], or inhibition [50] effect on soil microbial activities. The results of our study showed that the growth of vegetated patches has a positive effect on soil microbial communities. Indeed, the abundances of total PLFAs, bacteria, and fungi were significantly higher in inside-canopy soils than in outside-canopy soils and increased with shrub patch size. As shrub patch size increased, increases in GN bacteria were greater than increases in GP bacteria, which caused the ratio of GP to GN bacteria to decrease. High-nutrient environments favor the growth of r-strategy microbes, which are GN bacteria, and enhance nutrient cycling rates [51,52]. Higher concentrations of soil organic matter and total nitrogen might be linked to variations in fungal abundances and microbial biomass [53,54,55]. The main drivers of variation in soil microbial structure were changes in soil organic matter, total nitrogen, and available phosphorus, which is consistent with the results of previous studies in the middle Yellow River floodplain of China [29].

AMF PLFAs increased with shrub patch size, which is consistent with the results of previous studies [20,29]. Significant positive correlations between the soil salt content and AMF PLFAs suggest that islands of salinity provide substrates that promote the growth of AMF by the colonization of spores [56]. The soil salt content increased with shrub patch size at the two sampling locations. Increases in AMF can result in the generation of a large network of mycelia as the mycorrhizal roots extend, and this might have a positive effect on the nutrient uptake and soil organic matter accumulation of *T. chinensis* plants [57]. The growth of AMF can promote the formation and stabilization of soil aggregates and thus contribute to soil quality [11].

### 4.3. Implications

Vegetation restoration can promote increases in soil nutrients and soil quality [6,22,58]. In general, our study shows that *T. chinensis* plantations can enhance the content of soil nutrients and thus improve soil microbial community composition. The abundance of AMF increased with shrub patch size; increases in AMF promote plant growth and improve soil quality by altering the nutrient uptake capacity of plant roots [11,57]. Therefore, *T. chinensis* plantations could be planted in the middle Yellow River to promote ecological restoration efforts and improve the quality of the soil. Due to the one-time sampling in this research, the results may just be revealed as a snapshot, and further future long-term investigations concerning the lasting influence of *T. chinensis* plantations are required in this area.

## 5. Conclusions

The results of this study demonstrated that *T. chinensis* plants induced the enrichment of soil nutrients and microbial communities via the fertile island effect. Shifts in microbial community composition were mainly associated with changes in SOM, TN, and AP. The observed variation in soil microbial communities and soil nutrients suggests that *T. chinensis* could be used to promote ecological restoration efforts and alleviate the effects of land degradation in the middle Yellow River floodplain of China.

## Figures and Tables

**Table 1:** Shrub growth characters and particle size analysis in two sampling locations under small, medium, and large shrub patch sizes of *T. chinensis*.

Shrub Size	Sampling Location	Shrub Growth Characters (cm)			Particle Size Content (%)		
		Basal Diameter	Height	Canopy Diameter	Clay	Silt	Sand
Small	Inside	1.61 ± 0.13 C	147.25 ± 0.04 C	75.35 ± 3.40 C	5.54	65.61	28.85
	Outside				4.79	63.13	32.08
Medium	Inside	3.72 ± 0.13 B	262.75 ± 0.05 B	164.10 ± 6.46 B	4.59	63.65	31.76
	Outside				3.72	57.18	39.10
Large	Inside	8.89 ± 0.17 A	426.25 ± 0.08 A	275.12 ± 16.80 A	7.82	83.59	8.58
	Outside				7.36	85.68	6.96

Note: Different uppercase letters in the same column indicate significant differences at the 5% probability level.

**Table 2:** Soil physicochemical properties in the inside-canopy and outside-canopy soils under three shrub patch sizes of *T. chinensis*.

Shrub Size	Sampling Location	SWC (%)	pH	SSC (g·kg^−1^)	SOM (g·kg^−1^)	TN (g·kg^−1^)	AP (mg·kg^−1^)
Small	Inside	21.06 ± 0.74 Aa	8.12 ± 0.04 Aa	1.30 ± 0.17 Ab	4.28 ± 0.31 Ac	0.74 ± 0.03 Ab	44.37 ± 1.32 Ab
	Outside	20.42 ± 0.82 Aa	8.17 ± 0.02 Aa	0.89 ± 0.14 Aa	3.55 ± 0.34 Aa	0.72 ± 0.04 Aa	36.90 ± 1.72 Ba
Medium	Inside	20.98 ± 0.50 Aa	8.06 ± 0.04 Aa	1.36 ± 0.16 Ab	5.67 ± 0.55 Ab	0.80 ± 0.04 Ab	48.55 ± 2.55 Ab
	Outside	21.28 ± 0.50 Aa	8.15 ± 0.02 Aa	0.87 ± 0.10 Aa	3.63 ± 0.15 Aa	0.71 ± 0.01 Aa	38.99 ± 1.02 Ba
Large	Inside	21.96 ± 0.26 Aa	8.03 ± 0.04 Aa	2.43 ± 0.42 Aa	7.52 ± 0.27 Aa	1.02 ± 0.03 Aa	61.68 ± 2.03 Aa
	Outside	20.57 ± 0.58 Ba	8.09 ± 0.03 Aa	0.95 ± 0.19 Ba	4.17 ± 0.19 Ba	0.71 ± 0.03 Ba	39.89 ± 1.95 Ba

Note: All data are mean ± SE. SWC represents soil water content; pH represents soil Ph; SSC represents soil salt content; SOM represents soil organic matter; TN represents total nitrogen content; AP represents available phosphorus. Different uppercase letters indicate significant differences (*p* < 0.05) among sampling locations under the same shrub patch size; lowercase letters indicate significant differences (*p* < 0.05) among shrub patch sizes at the same sampling location (n = 4).

**Table 3:** Soil microbial PLFAs in the inside-canopy and outside-canopy soils under three shrub patch sizes of *T. chinensis*.

Shrub Size	Sampling Location	Total	Bacteria	Fungi	Fungi:Bacteria	GP Bacteria	GN Bacteria	GP:GN Bacteria	AMF
Small	Inside	46.61 ± 2.62 Ac	38.74 ± 2.34 Ac	7.87 ± 0.33 Ab	0.20 ± 0.01 Aa	9.59 ± 0.55 Ab	15.93 ± 1.33 Ab	0.61 ± 0.02 Aa	2.34 ± 0.14 Ac
	Outside	42.01 ± 1.64 Ab	36.12 ± 1.49 Ab	5.90 ± 0.23 Bb	0.16 ± 0.01 Bb	9.74 ± 0.56 Ab	14.79 ± 0.77 Ab	0.66 ± 0.01 Aa	1.29 ± 0.06 Bc
Medium	Inside	54.52 ± 0.82 Ab	46.13 ± 0.76 Ab	8.38 ± 0.23 Ab	0.18 ± 0.01 Ab	11.59 ± 0.27 Aa	19.05 ± 0.54 Ab	0.61 ± 0.02 Aa	3.57 ± 0.16 Ab
	Outside	51.94 ± 1.62 Aa	44.37 ± 1.42 Aa	7.57 ± 0.22 Ba	0.17 ± 0.01 Aab	10.15 ± 0.37 Bab	20.56 ± 0.71 Aa	0.49 ± 0.01 Bb	2.38 ± 0.11 Bb
Large	Inside	65.75 ± 1.54 Aa	55.63 ± 1.52 Aa	10.12 ± 0.18 Aa	0.18 ± 0.01 Ab	12.40 ± 0.37 Aa	24.49 ± 0.95 Aa	0.51 ± 0.01 Ab	5.32 ± 0.18 Aa
	Outside	54.10 ± 1.91 Ba	45.90 ± 1.73 Ba	8.20 ± 0.26 Ba	0.18 ± 0.01 Aa	11.04 ± 0.35 Ba	18.86 ± 1.39 Ba	0.59 ± 0.05 Aa	3.01 ± 0.08 Ba

Note: All data are mean ± SE. Total represents total PLFAs; Bacteria represents bacterial PLFAs; Fungi represents fungal PLFAs; Fungi:Bacteria represents the ratio of fungal to bacterial PLFAs; GP Bacteria represents Gram-positive bacterial PLFAs; GN Bacteria represents Gram-negative bacterial PLFAs; GP:GN Bacteria represents the ratio of Gram-positive bacterial PLFAs to Gram-negative bacterial PLFAs; AMF represents arbuscular mycorrhizal fungal PLFAs. Different uppercase letters indicate significant differences (*p* < 0.05) among sampling locations under the same shrub patch size; lowercase letters indicate significant differences (*p* < 0.05) among shrub patch sizes at the same sampling location (n = 4).

## Data Availability

Not applicable.

## Supplementary Materials

The following supporting information can be downloaded at: https://www.mdpi.com/article/10.3390/ijerph20065015/s1, Table S1: Results of two-way ANOVA analysis of the effects of shrub size (Size), sampling location (Location), and their interaction on soil physicochemical properties and soil microbial communities.
