# Supplementary material for: Effects of Variation in Tamarix chinensis Plantations on Soil Microbial Community Composition in the Middle Yellow River Floodplain
Source: Int J Environ Res Public Health. 2023 Mar 12;20(6):5015. doi: 10.3390/ijerph20065015 (PMC10049481; doi:10.3390/ijerph20065015)
Supplement: Supplementary file 1 [file ijerph-20-05015-s001.zip › ijerph-2149736-supplementary.pdf]

Table S1. Results of two-way ANOVA analysis of the effects of shrub size (Size), sampling location (Location), and their interaction on soil physicochemical properties and soil microbial communities.

Factors	Size		Location		Size × location	
	F	*P*	F	*P*	F	*P*
SWC	0.42	0.66	1.41	0.25	1.02	0.38
pH	3.20	0.07	5.76	**< 0.05**	0.18	0.84
SSC	4.54	**< 0.05**	18.94	**< 0.01**	3.58	0.05
SOM	17.80	**< 0.01**	58.79	**< 0.01**	8.04	**< 0.01**
TN	10.46	**< 0.01**	28.14	**< 0.01**	10.90	**< 0.01**
AP	16.04	**< 0.01**	74.54	**< 0.01**	8.90	**< 0.01**
Total PLFAs	38.97	**< 0.01**	18.73	**< 0.01**	3.60	**< 0.05**
Bacterial PLFAs	34.49	**< 0.01**	12.76	**< 0.01**	3.67	**< 0.05**
Fungal PLFAs	43.32	**< 0.01**	61.53	**< 0.01**	3.61	**< 0.05**
F/B ratio	0.84	0.45	13.64	**< 0.01**	5.85	**< 0.05**
GP bacterial PLFAs	11.68	**< 0.01**	6.44	**< 0.05**	2.20	0.14
GN bacterial PLFAs	21.13	**< 0.01**	4.62	**< 0.05**	6.55	**< 0.01**
GP/GN ratio	9.89	**< 0.01**	0.15	0.71	13.35	**< 0.01**
AMF PLFAs	170.78	**< 0.01**	212.66	**< 0.01**	14.98	**< 0.01**

Note: The bold numerals indicate the significance at $P < 0.05$.
